# Needle phobia in emergency department in a rural district hospital of Nepal: A cross-sectional analysis

**DOI:** 10.1371/journal.pmen.0000093

**Published:** 2025-01-03

**Authors:** Niraz Yadav, Aakriti Yadav, Neeru Yadav

**Affiliations:** 1 Terhathum District Hospital, Myanglung, Nepal; 2 National Academy for Medical Science, Kathmandu, Nepal; 3 Nepal Madhesh Foundation, Kupondole, Nepal; David Geffen School of Medicine: University of California Los Angeles David Geffen School of Medicine, UNITED STATES OF AMERICA

## Abstract

Needle phobia is a major concern for general practitioners in emergency department. Treatment of patients arriving for emergency care must be provided without delay. General practitioners face the challenge of managing patients with needle phobia, as it can exacerbate their condition, potentially leading to refusal of initial treatments. This emphasizes the importance of developing effective strategies to address needle phobia in emergency care. A cross-sectional study was conducted at Terhathum district hospital among 501 patients seeking treatment in the emergency department. Data were collected by face-to-face interview. This study reveals that 281 participants (56.2%) experienced needle phobia, with an average fear intensity score of 5.17 ± 1.45 on a 0–10 scale (0 indicating no fear and 10 indicating the highest intensity). The correlation of needle phobia with associated factors (p-value < 0.01) was analyzed by multivariate logistic regression. It was found that family history (AOR = 1.96, 95% CI = 1.33, 2.9), non-needle-related phobia (AOR = 1.88, 95% CI = 1.3, 2.72), and frequent need blood draw (AOR = 2, 95% CI = 1.36, 3.0) were significantly associated with needle phobia. The study indicates that 66% of individuals with needle phobia have a family history of phobia, compared to 49.5% of those without needle phobia, suggesting that both hereditary and environmental factors contribute to its development. Among various reason, general anxiety and pain were the two most common reasons for needle fear. Approximately 181 (64.4%) participants avoided medical treatment and out of those, 59 (32.6%) refused blood donation. Multiple response analysis revealed that relaxation techniques (26.7%), distraction during the procedure (22.1%), and education on how instruments work (16.7%) were the most effective methods for alleviating needle phobia. In conclusion, this study provides valuable insights into the prevalence, causes, and impact of needle phobia, emphasizing the importance of addressing it to improve patient experiences and compliance with medical treatments, leading to better health outcomes.

## 1. Introduction

Needles are frequently used medical devices to deliver effective management to sick patients. General practitioners working in emergency must recognize patients with needle phobia before administering injections of drugs, vaccines and many other essential procedures via a route of vascular, intradermal, subcutaneous and intramuscular routes. Needle phobia, a fear of medical procedures involving needles or injections, was officially termed Trypanophobia and reported in the Diagnostic and Statistical Manual of Mental Disorders (DSM-V) in 2013 under blood injection injuries [1]. Needle phobia is an anxiety disorder characterized by an unreasonable or irrational fear that significantly impacts daily living [1,2].

The prevalence of needle phobia varies according to studies conducted all over the world. A survey done in the US reported a very low prevalence (2.1%) in adults, [2] while another study conducted in the general adult population was found a prevalence of 63.2% [3]. A meta-analysis reviewing 119 original research articles concluded that 20–50% of adolescents and 20–30% in young adults have needle phobia [4]. Only a few children and adults can manage their fear of needles [5]. However, for some, the fear is so intense that it may prevent them from getting necessary injections, which can interfere with receiving proper healthcare.

It has been reported that people with trypanophobia often avoid having blood draws, taking insulin injections, canceling dentist appointments and even undergoing amniocentesis during pregnancy [4]. This avoidance is mainly due to significant portion of the population experiencing high levels of anxiety related to medical procedures [4,6–8]. The phobia is more frequent among the descendants of a phobic person, which points to the evidence of inheritance [9]. It is likely that the genetic form of needle phobia has an evolutionary basis, as humans who, thousands of years ago, meticulously avoided stab wounds and other instances of pierced flesh would have had a greater chance of survival [9]. Additionally, the fear of needles is often a learned behavior originating from childhood experiences [5].

Over time, multiple solutions have been proposed to address needle fear, based on associations between needle fear and various demographic, socio-economic and impact on health. Scientific innovations such as smaller/thinner needles and autoinjectors [10], as well as behavior changes and psychological counselling, have been adopted to counteract needle fear [8,11,12]. Our study aimed to investigate the prevalence, cause, impact and possible management of irrational fear of needle among the adult population presented in emergency department in a rural Terhathum district hospital of Nepal.

## 2. Materials and methods

### 2.1 Study design

A prospective cross-sectional study was conducted in rural primary-level government-owned hospitals in Nepal, using quantitative methods.

### 2.2 Study population

Our study population comprised patients present in the emergency department during the study period from December 24^th^ 2023, to February 24^th^ 2024. General practitioners in the emergency department must provide necessary medication via needle insertion for cannulation, local anesthesia and vaccination for initial treatment. Thus, a total of 501 patients who had undergone needle insertion and were able to complete given pre-structured questionnaires were included in the study period. Samples were collected from participants before and after the needle procedure by convenience sampling method and all participants signed informed consent forms. Participation in the study was free and no incentive was provided. Patients who were unable to complete the questionnaires and aged less than 18 years were excluded from the study.

### 2.3 Study site

The study site was Terhathum district hospital, situated in Myanglung town in Koshi province, Nepal. Terhathum district hospital serves people living in difficult hilly areas and is the only primary level hospital in the region that is well-equipped with capable human resources. The nearest tertiary referral center is about four to five hours away from town. At a given setting more information can be attained since people attending hospitals have diverse ethnicities, cultures and lifestyles. The triage process implemented at the emergency department at Terhathum district hospital follows the same protocol as the largest hospital in Nepal, Tribhuvan University Teaching Hospital (TUTH), adhering to the triage guidelines set by the World Health Organization (WHO) [13]. According to these guidelines, the emergency department must have three separate areas based on the decreasing order of severity. Patients in red areas are those who cannot survive without immediate treatment, followed by patients in the yellow area who need to be observed frequently (and possibly later re-triage) as their condition is stable. The green areas are reserved for the least of severity.

### 2.4 Sample size and sampling method

The total sample size was 501. The face to face interview was conducted from 24^th^ December 2023 to 24^th^ February 2024. The sample size was calculated from the Cochran formula (n) = z^2^pq/d^2^, assuming the prevalence (p) of needle phobia as 27.4% from a previous study conducted [14], at statistics corresponding to the level of 95% confidence interval (z), 4% tolerable error (d) and 5% non-response rate (q).

### 2.5 Data collection tools and techniques

Data was collected through face-to-face interviews with adult patients who came for treatment and were admitted to the yellow triage area in emergency department. Our questionnaires were adapted from a study titled “Prevalence, causes, impacts and management of needle phobia: an international survey of a general adult population” [3] with permission granted via email with the condition of inclusion of a copyright statement on all copies. All the author’s contact details were provided at the bottom of the questionnaires. Certain questions, such as those related to "device-based strategies" and "geographical region," were excluded from our questionnaires. Additionally, household income categories were modified to reflect paid or unpaid professions and IPS-Anx (Injection phobia scale-Anxiety) were included in questionnaire.

Participants were selected by convenience sampling method. Detailed information about the study was shared with patients, and a written formal consent was taken. Questions were available in both Nepali and English, allowing participants to choose their preferred language for responses. The translated questionnaire was pilot tested with 50 participants to ensure that the items were clearly understood. The questions were categorized into four groups:—(1) Sociodemographic information of patients (2) IPS-Anx (Injection phobia scale-Anxiety) (3) Cause and Impact and (4) Management. The IPS-Anx scale comprises 18 items rated on a five-point Likert scale, ranging ranges from 0 (no anxiety) to 4 (most anxious). The factor structure, reliability and validity of the IPS-Anx were confirmed by Olatunji et al [7]. Initially questions were asked up to the third group, focusing on cause and impact. Following this, the pain tolerance score based on previous experiences was recorded. Counseling was then provided on strategies to cope with needle fear. Patients were allowed to experience needle pricks in various forms, such as injections, cannulation and sutures depending upon treatment required. After that, questions from the fourth group on highest effective management and priority-based rank method were asked and recorded, followed by any improvement or lack of improvement in the pain tolerance score. Thus data were collected before and after the medical procedures to capture patients’ actual clinical responses to questions regarding their needle-related fears.

### 2.6 Statistical analysis

Data was entered in EpiData version 3.1. After confirming the completeness, data were exported to IBM SPSS Version 23 for further analysis. Univariate analysis was explored using frequencies, percentages, mean, mode, median and standard deviation. Multicollinearity was assessed with variance inflation factor (VIF) values for all independents variables was set not greater than 5 [15]. The goodness of fit, regression model was tested by applying Hosmer and Lemeshow chi-square test. Those variables significantly associated in the univariate analysis at 95% confidence level, P-value less than 0.01 were included in the multivariate model. We applied multivariate logistic regression analysis adjusting for covariates such as family history, non-needle-related fear and frequent blood draws to identify the factors associated with needle phobia. The unadjusted and adjusted odds ratio with 95% confidence interval was reported. P-value less than 0.01 was considered statistically significant. Continuous variables were summarized using mode, median and interquartile range. The participants’ responses to the ranking questionnaires with given options were tabulated in the "Multiple Response" section of the analysis using the "Define Set Variable" function in SPSS.

### 2.7 Ethics consideration and confidentiality

A written administrative approval was obtained from the Chief of Terhathum district hospital (Approval No.202). Subsequently, ethical approval was granted by the Ethical Review Board of the Nepal Health Research Council (Approval No: 899). The participants signed a formal written consent, and the authors personally collected all the data, and also ensured that each participant was coded during data collection to maintain confidentiality.

## 3. Result

### 3.1 Background characteristics of the study participants

The result included 501 participants, who had attended emergency care in district hospital. The mean (+SD) age of participants were 43±1.5 years ranging from 18 years to 94 years. There were 269 (53.7%) female participants, while 274 (54.7%) were belong to non-advantaged ethnicity. More than half of participants, 271 (54.1%) had an education level of secondary and above. Nearly, two thirds of participants 369 (73.8%) had paid jobs out of which very few 68 (13.6%) had currently or previously worked as a healthcare professionals. A total of 180 (36%) had a positive history of needle phobia in the family while evidence of non-needle-related medical fears were 285 (57%). During medical procedures 216 (43.2%) had intravenous route, 150 (29.8%) intramuscular route, 66 (13.2%) intradermal and 69 (13.8%) had others forms of injections. Among those 188 (37.6%) were frequently required blood draws. The prevalence of needle phobia revealed 281 (56.2%) with a notably higher prevalence among females 160 (59.5%) compared to males 121 (52%). Here is a more structured and detailed description in Table 1, which outlines the sociodemographic characteristics of the research participants.

**Table 1:** Sociodemographic information of participants.

SN	Sociodemographic factors	Total participants		Participants with fear of needle				
		501		Yes (281)		No(220)		P-value
		N	%	N	%	N	%	
1.	**Age group(years)**							
	18 to 24	80	16%	67	84%	13	16%	0.00*
	25 to 34	85	17%	63	74%	22	26%	
	35 to 44	82	16.4%	45	55%	37	45%	
	45 to 54	84	16.8%	39	46%	45	54%	
	55 to 64	78	15.6%	30	38%	48	62%	
	65 to 74	60	12%	29	48%	31	52%	
	75 and above	32	6.2%	8	25%	24	75%	
2	**Sex**							0.1
	Male	232	46.3%	121	52%	111	48%	
	Female	269	53.7%	160	59.5%	109	40.5%	
3	**Ethnicity**							0.013
	Advantaged	227	45.3%	141	62%	86	38%	
	Non-advantaged	274	54.7%	140	51%	134	49%	
4	**Level of education**							0.19
	Primary and below	230	45.9%	142	61.7%	88	38.3%	
	Secondary and above	271	54.1%	139	51%	132	49%	
5	**Profession**							0.545
	Paid	369	73.8%	204	55.3%	165	44.7%	
	Unpaid	132	26.2%	77	58.3%	55	41.7%	
6	**Current and previous work as healthcare professional**							0.039
	Yes	68	13.6%	46	67.6%	22	32.4%	
	No	433	86.4%	235	54.3%	198	45.7%	
7	**Needle phobia family history**							0.001*
	Yes	180	36%	119	66%	61	34%	
	No	321	64%	162	50.5%	159	49.5%	
8	**Any non-needle-related medical fears**							0.002*
	Yes	285	57%	177	62%	108	38%	
	No	216	43%	104	48%	112	52%	
9	**Condition that require frequent injection or blood draw**							0.001*
	Yes	188	37.6%	124	66%	64	34%	
	No	313	62.4%	157	50.2%	156	49.8%	
10	**Route of administration**							0.59
	IV	216	43.2%	119	55.1%	97	44.9%	
	IM	150	29.8%	80	53.3%	70	46.7%	
	Others	69	13.8%	43	62%	26	38%	
	ID	66	13.2%	39	59%	27	41%	

Note: *P- Value 0.01 is significant.

### 3.2 Assessment of needle phobia

The findings revealed more than half of the participants had needle phobia 281 (56.2%). Among them, half of the participants had a fear of needles during medical procedures 144 (51.2%) and 113 (40.2%) had before a medical procedure. In addition, the participants experiencing needle phobia were administered the Standard Injection phobia scale for Anxiety (IPS-Anx) [3,7,8,11]. Table 2 outlines the invasiveness of procedures and the associated anxiety levels, revealing that techniques involving direct needle contact, which provoked anxiety as shown in the contact fear items, had the highest mode and median values, particularly for invasive items like "Having an anesthetic injection at the dentist’s" and "Having a venipuncture." Both items reached a median and mode of 3 while distal fear appeared in scenarios where participants felt anxious merely by observing a needle from a distance, without direct contact.

**Table 2:** Injection phobia scale-Anxiety (IPS-Anx).

SN	Questions	IQR	Median	Mode
	**Items of contact fear**			
1	Giving a blood sample by having a finger pricked	1–3	2	2
2	Having a shot in the upper arm	1–3	2	2
5	Having an anesthetic injection at the dentist’s	1–4	3	3
6	Having a venipuncture (needle inserted into vein)	1–3	3	3
8	Getting an injection in the buttock	1–3	2	2
15	Having one’s ears pierced	0–2	2	2
16	Getting a vaccination	0–2	2	2
17	Getting an intravenous injection	1–3	2	2
	**Items of distal fear**			
3	Looking at a picture with a syringe and needle	0–1	1	1
4	Sensing the smell of a hospital	0–1	0	0
7	Watching another person having a venipuncture in reality	0–2	1	1
9	Looking at a picture of a person getting a shot	0–1	0	0
10	Listening to someone talking about injections	0–1	0	0
11	Looking at and touching veins in the crook of the arm	0–1	0	0
12	Watching a film about a person getting a shot	0–1	0	0
13	Watching another person getting a shot in reality	0–2	1	1
14	Watching a person in a nurse uniform	0–1	0	0
18	Watching another person having a finger pricked in reality	0–2	0	0

Note: IQR = inter quartile range.

The highest contributor of needle phobia was found to be general anxiety 71 (25.3%) and second is pain 60 (21.4%). In total 181 (64.4%) avoided medical treatment at some point in life and the main reason was fear of something going wrong during procedure 101 (55.8%) and second was pain 92 (50.8%). Despite of fear 122 (67.4%) agreed while 59 (32.6%) refused to donate blood as shown in Table 3. In total 174 (61.9%) had fear to needle since childhood (<18 years).

**Table 3:** Causes and impacts of needle fear in Terhathum district hospital.

Causes and Impacts of Needle Fear	Frequency	Percentage
**Highest contributor of Needle Phobia (281)**		
General anxiety	71	25.3%
Pain	60	21.4%
Fear something going wrong during procedure	51	18.1%
Previous traumatic experience with needle	45	16%
Having to see blood	21	7.5%
Fear of fainting/ feeling dizzy	16	5.7%
Other	10	3.6%
Disgust regarding the procedure	7	2.5%
**How long had needle phobia (281)**		
<18years	174	61.9%
>18years	107	38.1%
**Avoided medical treatment (281)**		
Yes	181	64.4%
No	100	35.6%
If yes, main reason to avoid procedure (181)*		
Fear for something would go wrong during procedure	101	55.8%
Pain	92	50.8%
Previous traumatic experience with needle	82	45.3%
General anxiety	78	43.1%
Having to see blood	53	29.3%
Fear of fainting/feeling dizzy	13	7.2%
Others	12	6.6%
**If yes, would you Blood donation (181)**		
Yes	122	67.4%
No	59	32.6%
**Which of the following procedures would you avoid in order to reduce your exposure to needles?(281)** *		
Capillary blood draw(finger prick)	162	57.6%
Vaccination	159	56.6%
Blood donation	89	31.7%
Blood draw from vein	77	27.4%
Injection for pain relief	48	17.1%
Injection for the treatment of a medical condition	36	12.8%

Note: *Participants would select more than one option.

**Fig 1:**
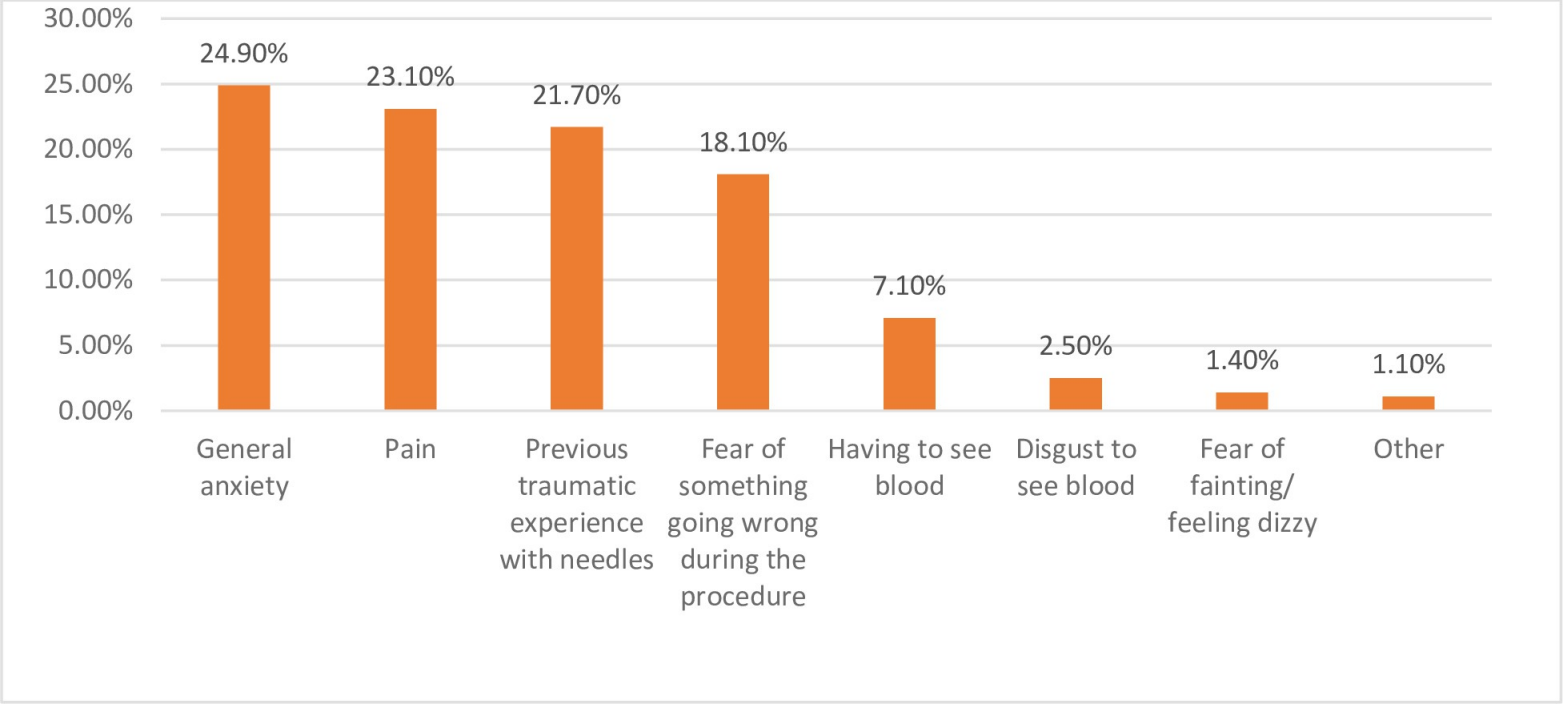
Multiple response chart showing cause of needle phobia.

Fig 1 illustrates the detailed causes of needle phobia. Participants were asked to rank one to eight on different variables in order of significance. Then data were analyzed using multiple responses and set variables in SPSS. The analysis revealed that the most significant causes of needle phobia are general anxiety (24.9%), pain (23.1%), previous traumatic experiences (21.7%) and fear of something going wrong during the procedure (18.1%). These four factors were identified as the top contributors, while other variables were least relevant basis of cause in causing needle phobia.

On giving opinion to reduce exposure to needles majority of participants would like to avoid capillary blood draw (finger prick) 162 (57.6%), vaccination 159 (56.6%) followed by blood donation 89 (31.7%). During procedures in the emergency department, most of the participants said educating on how instruments work 66 (23.5%) and relaxation techniques like the deep breathing technique 65 (23.1%) were most helpful to reduce fear of needles. Distraction 62 (22.1%) during procedures were almost equally helpful. Watching blood draw videos before/during the procedure 15 (5.3%) were least effective method to reduce fear. A detailed document has been presented in Table 4. Nearly 203 (72.2%) had never sought help for needle phobia. On average, when rating from 0 to 10 (0 is no fear and 10 being highest/unreasonable fear or avoidance), participants had a pain tolerance score of 5.17 ± 1.45 before the needle procedures. After receiving education and counseling on methods to reduce fear of needles, the medical procedure/intervention was performed, and their final pain tolerance improved to 5.45 ± 1.32.

**Table 4:** Management of needle phobia using non-device-based strategies in rural districts of Nepal.

**Most helpful methods to reduce fear of needle(281)**		
Education on procedure instruments	66	23.5%
Relaxation technique like deep breath technique	65	23.1%
Distraction during procedure	62	22.1%
Consultations with clinician regarding importance of procedure	39	13.9%
Noninvasive (use of topical cream)	34	12.1%
Watching blood draw videos before/during the procedure	15	5.3%
**Have you ever sought help for needle phobia(281)**		
No	203	72.2%
Yes, took other action	66	23.5%
Yes-saw a therapist	12	4.3%

**Fig 2:**
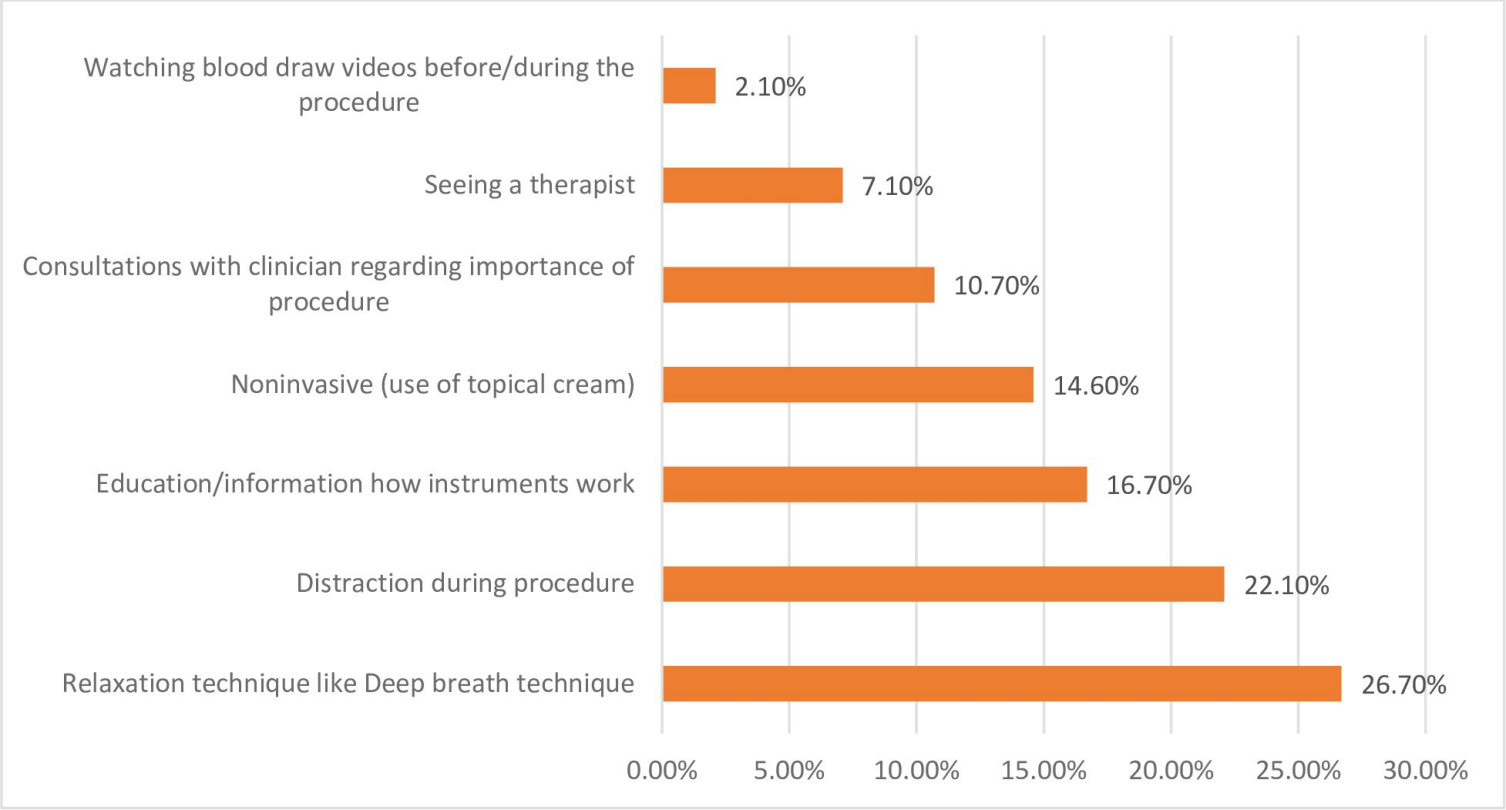
Ranked chart of management of needle phobia.

On ranking the effective methods to alleviate needle fear among participants, we again employed multiple responses in which relaxation technique like deep breath technique (26.7%) were the most while distraction during procedure (22.1%) and educating/ sharing information on how instruments work (16.7%) were second and third respectively, improved patient comfort and cooperation in medical settings. Seeing a therapist (7.1%) and watching blood draw videos before /during the procedure (2.1%) were the least effective methods of reducing fear of needles. The detail is presented in Fig 2.

### 3.3 Factors associated with needle phobia

According to this study finding, the regression analysis showed that the risk of needle phobia were more likely for candidates who had family history (AOR = 1.96, 95% CI = 1.33, 2.9), non-needle related phobia (AOR = 1.88, 95% CI = 1.3, 2.72) and participants who frequently required blood draw had experience twice to fear with needle (AOR = 1.99, 95% CI = 1.36, 2.93) as shown in Table 5.

**Table 5:** Factors associated with needle phobia in rural districts of Nepal.

Variables			Unadjusted OR (COR)	95% CI	P-value	Adjusted OR (AOR)	95% CI	P-value
**Family history**								
Yes	180	36%	1.91	1.31,2.79	0.001	1.96	1.33,2.9	0.001
No	321	64%	1			1		
**Frequently require blood draw**								
Yes	188	37.6%	1.92	1.32,2.79	0.001	2	(1.36,3.0)	0.000
No	313	62.4%	1			1		
**Non needle phobia**								
Yes	285	57%	1.76	1.23,2.52	0.002	1.88	(1.3,2.72)	0.001
No	216	43%	1			1		

Note:*Statistically significant (p<0.01) at 95% CI, COR = Crude Odds Ratio, AOR = Adjusted Odds Ratio.

## 4. Discussion

Our study described the detailed preferences and perspectives regarding needle phobia among people living in rural hilly region of Nepal. The prevalence of needle fear was found to be as high as 56.2% which is significantly higher than the 20–30% reported in a systemic review analyzing 119 research papers from different countries [4]. The prevalence is notably lower than study conducted in a different part of the world [3] and higher than 27.4% reported among Jordanian adults [14], 39% of adults attending one of two inner-city travel clinics [16] and 30.9% in medical student in India [8]. Our findings also indicate that needle fear decreases with age, dropping to 3% among the elderly. This trend aligns with findings from several other studies [3–5]. Additionally, we concluded that females were more likely to experience needle phobia compared to males which is consistent with other research [3,4,8,10,14]. The variations in needle phobia prevalence could be attributed to differences in study settings and the demographic and socioeconomic characteristics of the study participants. Age, in particular, is considered a key factor influencing needle phobia.

Needle phobia can be influenced by genetic predisposition [9], environmental factors, and past traumatic experiences [10,17,18]. The observation that 66% of phobic patients had a family history suggests a potential hereditary component to phobia development. However, 49.5% of non-phobic individuals also reported a family history indicating that environmental factors may play a significant role as well. This emphasizes the complexity of phobia development, where genetic predisposition could interact with environmental triggers, such as traumatic experiences or learned behaviors, to influence the likelihood of developing a phobia. Thus, understanding this interplay is crucial for developing effective prevention and treatment strategies.

Approximately 25.3% of participants in our study identified anxiety as the primary contributor to their fear of needles, a finding consistent with several other studies [3,4,7,8,11,16,19]. Moreover, our study highlighted that the Injection Phobia Scale-Anxiety (IPS-Anx) demonstrates excellent psychometric properties, indicating its suitability for use in research focused on injection phobia [7]. Pain is another significant factor contributing to needle phobia, which can have adverse effects on individuals’ health. Many participants in our study reported avoiding medical treatments due to their fear of needles [9,10,16,19,20]. This avoidance behavior was often driven by a gut feeling that something would go wrong during the procedure, pain experienced during needle insertion, and previous traumatic experiences. These findings are supported by other studies that have shown individuals screening positive for needle phobia are more likely to express vaccine hesitancy [19], avoid chemotherapy (17–52%), and refuse blood draws (52.2%) [3,10]. Consequently, needle phobia encompasses a complex interplay of anxiety, pain sensitivity, and past trauma, significantly impacting healthcare-seeking behaviors and potentially compromising health outcomes.

On multivariate logistic regression analysis, using the goodness of fit test by Hosmer and Lemeshow chi-square test, several factors were significantly associated with the risk of developing needle phobia at a P-value < 0.01. These factors included a family history of needle phobia, frequent requirements for blood draws, and non-needle related phobias. The influence of family history on needle phobia has been noted in previous research, such as the Mayo Clinic study in 2001, which reported that approximately 80% of individuals with needle phobia had first-degree relatives who also exhibited a similar fear. This higher correlation in family history appears to be prevalent in the general adult population, potentially influenced by cultural factors where fear of needles or injections is learned from parents. Parents’ warnings or cautionary tales about needles during childhood may contribute to the persistence of fear into adolescence and adulthood, impacting healthcare experiences. Of interest, an analysis conducted in Australia did not support such high statistics regarding the influence of family history on needle phobia [10]. This discrepancy may reflect variations in cultural attitudes towards medical procedures and fear responses across different populations.

Our study, observed a strong correlation between the frequency of required blood draws (frequent needle pricks) and the fear associated with them, which is consistent with findings from other research [3]. For instance, patients undergoing hemodialysis, who may require up to 312 needle insertions per year, reported needle fear rates ranging from 25% to 47% [10]. Similarly, adolescents treated with multiple daily insulin injections (MDI) were found to be at significant risk for developing a fear of self-injection of insulin, which often led to avoidance of necessary medical procedures [21]. Among participants in our study who reported avoiding medical procedures due to fear, the primary reasons included a fear that something would go wrong during the procedure, followed by concerns about pain. Of interest, despite their fear of needles, a majority of participants expressed willingness to donate blood if needed. When asked about methods to reduce exposure to needles, many participants preferred capillary blood draws (fingersticks) and vaccinations as preferred options. Furthermore, participants highlighted that medical equipment-related education, deep breathing techniques, and distraction were the most helpful non-device-related strategies to reduce fear. These findings are supported by existing literature, which suggests that non-device techniques are effective measures against needle phobia [3,8,11]. Moreover, our study examined the impact of psycho-education provided to participants in the yellow triage of an emergency department, aimed at increasing overall pain tolerance. The results showed a statistically significant improvement, with the mean pain tolerance score increasing from 5.17 ± 1.45 to 5.45 ± 1.32 after the intervention. This underscores the effectiveness of targeted psycho-education in enhancing pain tolerance among individuals with needle phobia.

### 4.1 Limitations and strengths

Since our study is from the rural Terhathum district, our results cannot be generalized to the population of Nepal due to potential differences in demographics, healthcare access and cultural factors. For example, a study conducted among medical students in an urban medical college in Nepal had different experiences about needle fear [22]. Further, it is important to note that our study did not include questions about "device-based strategies." This limitation could affect the comprehensiveness of our findings, especially considering advancements in medical technology and alternative methods for administering treatments.

## 5. Conclusion

Based on the study’s findings from the Terhathum district hospital, it’s evident that needle phobia is prevalent among adult patients attending for emergency care, particularly among females. Several factors were identified as significantly associated with needle phobia, including family history, frequent blood draws, and non-needle related phobias. The implications of these findings suggest that there is a substantial need for national preventive programs to address needle phobia among patients seeking emergency treatment. Needle phobia not only complicates medical procedures by potentially avoiding necessary treatments but also exacerbates symptoms in patients who reluctantly agree to needle insertions. Moreover, the study highlights the necessity for more detailed research and analytical methods to bridge gaps in the scientific literature and develop effective treatments for needle phobia. Improving the medical experience for patients through these efforts could significantly enhance treatment adherence and outcomes in emergency settings.
